# Caspase-8 tyrosine-380 phosphorylation inhibits CD95 DISC function by preventing procaspase-8 maturation and cycling within the complex

**DOI:** 10.1038/onc.2016.99

**Published:** 2016-04-25

**Authors:** I R Powley, M A Hughes, K Cain, M MacFarlane

**Affiliations:** 1 MRC Toxicology Unit, Leicester, UK

## Abstract

Caspase-8 is a key initiator of apoptotic cell death where it functions as the apical protease in death receptor-mediated apoptosis triggered *via* the death-inducing signalling complex (DISC). However, the observation that caspase-8 is upregulated in many common tumour types led to the discovery of alternative non-apoptotic, pro-survival functions, many of which are contingent on phosphorylation of a tyrosine residue (Y380) found in the linker region between the two catalytic domains of the enzyme. Furthermore, Src-mediated Y380 phosphorylation leads to increased resistance to CD95-induced apoptosis; however, the mechanism underlying this impaired response to extrinsic apoptotic stimuli has not been identified. Consequently, we have employed a number of model systems to further dissect this protective mechanism. First, using an *in vitro* DISC model together with recombinant procaspase-8 variants, we show that Y380 phosphorylation inhibits procaspase-8 activation at the CD95 DISC, thereby preventing downstream activation of the caspase cascade. Second, we validated this finding in a cellular context using transfected neuroblastoma cell lines deficient in caspase-8. Reconstitution of these lines with phosphomimetic-caspase-8 results in increased resistance to CD95-mediated apoptosis and enhanced cell migration. When the *in vitro* DISC is assembled in the presence of cell lysate, caspase-8 Y380 phosphorylation attenuates DISC activity by inhibiting procaspase-8 autoproteolytic activity but not recruitment or homodimerization of caspase-8 within the complex. Once incorporated into the DISC, phosphorylated caspase-8 is unable to be released from the complex; this inhibits further cycling and release of active catalytic subunits into the cytoplasm, thus resulting in increased apoptotic resistance. Taken together, our novel findings expand our understanding of the key mechanisms underlying the anti-apoptotic functions of caspase-8 which may act as a critical block to existing antitumour therapies. Importantly, reversal or inhibition of caspase-8 phosphorylation may prove a valuable avenue to explore for sensitization of resistant tumours to extrinsic apoptotic stimuli.

## Introduction

Caspase-8 has been well characterized as an initiator of the extrinsic pathway of apoptosis. However, more recent studies have revealed that it also has alternate, non-apoptotic, functions.^1^ As an initiator caspase, procaspase-8 is recruited to the cell-surface membrane *via* members of the tumour necrosis factor (TNF) receptor superfamily such as CD95 (Fas/Apo1) and TNF-related Apoptosis-Inducing Ligand Receptors 1 and 2 (TRAIL-R1 and TRAIL-R2).^2^ These family members possess a conserved intracellular cysteine-rich Death Domain (DD), and ligand-induced receptor aggregation of these DDs results in the recruitment of the DD-containing adaptor molecule, Fas-Associated protein with Death Domain (FADD), through homophillic DD/DD interactions. In addition, FADD also possesses an N-terminal death effector domain (DED) responsible for the recruitment of procaspase-8 to the nascent complex. The assembly of activated receptor, FADD and procaspase-8 is collectively termed the death-inducing signalling complex (DISC). However, once at the DISC, caspase-8 is only fully activated following a two-step process involving the initial tethering of a procaspase-8 dimer; at this juncture, caspase-8 displays only low intrinsic catalytic activity and a narrow substrate range.^3^ For full activity, a second proteolytic step is required to remove the linker region connecting the large and small catalytic subunits; this now fully activated DISC is capable of cleaving a far greater range of substrates, including caspase-3 and Bid, and is thus capable of driving apoptotic cell death.^3, 4^

In addition to its apoptotic role, caspase-8 has been shown to have many disparate roles in alternative cellular processes, completely separate to those involved in apoptosis. For example, both CD95 and TNF-R1 are capable of signalling for cell survival *via* activation of non-apoptotic pathways involving NFκB and MAP kinases, as well as regulating neutrophil survival.^1, 5, 6, 7^ It has also become increasingly clear that both caspase function and activity can be critically regulated by phosphorylation of key residues within the protein. Indeed, the activity of caspases-2, -3, -9 and -8 have all been demonstrated to be modified by phosphorylation.^8^

Caspase-8 itself has been shown to be phosphorylated on multiple residues, including Tyrosine-293, -380 and -448 as well as serines -287, -305 and -347.^9, 10, 11^ To date, the majority of caspase-8 phosphorylation-focused studies have concentrated on Tyr380 (Y380), located in the linker region joining the large and small catalytic subunits. Phosphorylation of this residue can be effected by Fyn, Lyn and Src kinases and potentially dephosphorylated by the phosphatase SHP1.^7, 9^ Thus, Y380 phosphorylation could potentially have a critical role in cancer progression and metastasis as its phosphorylation targets caspase-8 to membrane ruffles, independently of its catalytic activity, promoting migration as well as PI3K-mediated endosome maturation *via* interaction with the p85α subunit of PI3K.^12, 13^ Furthermore, phosphorylation of caspase-8 at Y380 leads to increased resistance to CD95-mediated apoptosis in a number of tumour cell lines and colon cancer.^14^ This decreased rate of apoptosis appears to be largely due to the reduced rate of caspase-8 processing as evidenced by reduced p18 formation upon treatment with FasL/CD95L following epidermal growth factor (EGF)-induced phosphorylation of caspase-8.^14^ Moreover, structural studies performed on truncated caspase-8 variants lacking the DEDs revealed that Y380 phosphorylation results in a lowered rate of caspase-8 processing and preferential cleavage of non-phosphorylated caspase-8 over that of the phosphorylated form.^15^

To date, many of the studies investigating caspase-8 function have relied on truncated variants, lacking the DED domains of the inactive zymogen, and thus have ignored the potentially important contributory roles that these domains play in modulating caspase-8 function.^15^ Indeed, the importance of both DED domains was highlighted by our recent discovery of the critical role for DED-mediated procaspase-8 chain assembly in TRAIL DISC-driven apoptotic cell death.^16^ In this study, we have further characterized the role of caspase-8 phosphorylation in regulating its catalytic activity, cell migration and cell survival signalling utilizing full-length procaspase-8 activated within its native cell death signalling complex. Furthermore, we show the effects of Y380 phosphorylation on the NFκB signalling pathway, migration and cell death. Using an *in vitro* CD95 DISC reconstitution model and native DISCs isolated from neuroblastoma cell lines, we have directly assessed the effects of procaspase-8 phosphorylation on DISC activation and its downstream activity. We now show that when the *in vitro* DISC is assembled in the presence of cell lysate, Y380 phosphorylation blocks complete procaspase-8 maturation and cycling at the DISC by inhibiting its autoproteolytic activity. However, critically, phosphorylation does not prevent DISC recruitment or homodimerization of procaspase-8, rather it is the inhibition of auto-catalytic processing that prevents release of the mature caspase-8 catalytic subunits and inhibits subsequent activation of the downstream caspase cascade.

## Results

### Effect of caspase-8 phosphorylation on downstream signalling pathways

**Figure 1:**
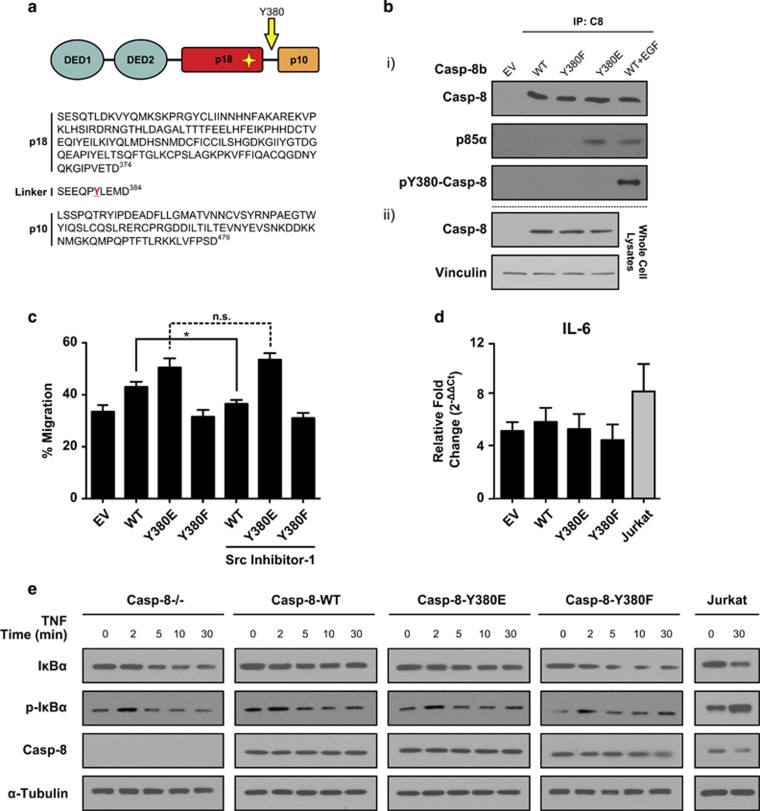
Caspase-8 (Y380E) mutant mimics constitutive phosphorylation. (**a**) Primary-amino acid sequence of caspase-8 catalytic subunits indicating the putative phosphorylation site present in the linker region in red. Mach-α1 (Caspase-8a) numbering is used throughout. (**b**) (i) SH-SY5Y cells expressing caspase-8 with Tyr (WT), Glu (Y380E) or Phe (Y380F) at amino acid 380 were immunoprecipitated with an anti-caspase-8 antibody and protein A/G beads and immunoprecipitated samples analysed by SDS–PAGE and immunoblotted for p85α, total caspase-8 or phospho-Tyr380-casp-8b. Wild-type caspase-8 phosphorylation was induced by a 30 min treatment with EGF (25 ng/ml). (ii) Protein expression levels of caspase-8 in SH-SY5Y cells expressing caspase-8 WT, Y380E or Y380F variants show equal expression across the reconstituted cell lines. (**c**) SH-SY5Y cells overexpressing caspase-8 were grown to confluence on a fibronectin substrate, the monolayer wounded and the percentage migration into the wound measured after 24 h. Data shown are the mean+s.e.m. of *n*=3. The effect of Src inhibition was investigated by 16 h pre-treatment with Src-Inhibitor-1 (5 μm). *P<0.05; n.s. denotes not statistically significant. (**d**) Wild-type Jurkat cells (clone A3) or SH-SY5Y cells overexpressing caspase-8b variants were incubated with TNFα (50 ng/ml) for 6 h and quantitative RT–PCR expression analysis of the NFκB target gene IL-6 measured relative to Ribosomal Protein L18. The fold-change in TNF-treated cells relative to untreated matched control cells was calculated using the ΔΔCt method. Data shown are mean of triplicate repeats+upper range. No significant difference was observed between the SH-SY5Y cell lines. Jurkat cells served as a positive control. (**e**) Wild-type-Jurkat cells or SH-SY5Y cells overexpressing caspase-8b variants were incubated with TNFα (50 ng/ml) for up to 30 min and cells harvested at the time points indicated. Levels of caspase-8, total and phosphorylated IκB and tubulin were assessed by immunoblotting.

It has become increasingly clear that caspase-8 has a secondary function in many traditionally non-canonical roles; these include cell migration, metastasis and cell survival signalling *via* the NFκB pathway. Although caspase-8 phosphorylation at Tyrosine-380 has been reported to be involved in some of these processes,^5, 13, 14^ it had not previously been addressed whether caspase-8 phosphorylation similarly has a corresponding role in NFκB signalling. To investigate this, we generated caspase-8-deficient SH-SY5Y neuroblastoma cells overexpressing equivalent amounts of wild-type procaspase-8b (Casp-8b-WT) and mutant variants, namely Casp-8b-Y380E and -Y380F; which mimic constitutive phosphorylation at Tyr380 by replicating its charge characteristics and prevent phosphorylation at this site, respectively (Figures 1a and b).

To confirm that the Casp-8b-Y380E mutant was indeed mimicking constitutive phosphorylation, we took advantage of prior reports which have indicated that phosphorylation of caspase-8 at this position leads to its association with the p85α regulatory subunit of PI3K and subsequent modulation of endosomal maturation.^12, 13^ Thus, we hypothesized that caspase-8 Y380E should also associate with p85α. To test this, immunoprecipitations were performed using an antibody against caspase-8. In both the unstimulated wild-type and non-phosphorylatable Casp-8b-Y380F mutant cell lines, p85α did not associate with caspase-8. However in both the Casp-8b-Y380E expressing cells and in samples where caspase-8 phosphorylation was induced upon EGF stimulation, p85α co-immunoprecipitated with caspase-8 (Figure 1b), thus mimicking the native phosphorylation.

As caspase-8 has been implicated in promoting cell migration *via* localization to the cell membrane, we next investigated whether the Y380E mutation could modulate cell migration. When assessed in a wound healing assay, cells overexpressing the non-phosphorylatable Casp-8b-Y380F variant displayed reduced wound healing ability compared with the wild-type sample (Figure 1c); conversely, overexpression of the Y380E variant promoted cell migration. As previous studies had implicated Src as one of the key kinases responsible for caspase-8 phosphorylation,^14^ we tested whether addition of a Src inhibitor could reduce or prevent cell migration. Thus, we assayed cell migration after a 16h pre-treatment with Src-inhibitor-1, a selective, competitive inhibitor of the Src family of tyrosine kinases. Importantly, the migratory rate of both the Casp-8b-Y380E and Casp-8b-Y380F variants remained unaltered following Src inhibition; however, cells expressing wild-type caspase-8 showed significantly reduced wound healing capacity. Together, these data further support the role of Src-mediated caspase-8 phosphorylation in cell migration and metastasis.

Given that caspase-8 phosphorylation is an important factor in deciding cell fate following exposure to extrinsic apoptotic stimuli, we assessed whether caspase-8 phosphorylation altered pro-survival signalling *via* TNF-induced NFκB activation. As NFκB activation is preceded by the phosphorylation and subsequent degradation of its cellular inhibitor IκBα, measurement of the phosphorylation and loss of this protein can be used as an indicator of NFκB activation. Therefore, we treated the various cell lines with TNFα (50 ng/ml) for up to 30 min and followed time-dependent alterations in IκBα levels and phosphorylation status, and further quantified the NFκB-mediated induction of IL-6 mRNA by qRT-PCR at 6 h post-treatment. In all cell lines there was a rapid increase (within 2 min) in the phosphorylation of IκBα upon TNF treatment, followed by its rapid degradation within 10 min and subsequent induction of IL-6 mRNA. However, there was no significant difference between the various cell types in either case (Figures 1d and e). Hence, it appears that caspase-8 phosphorylation at Tyrosine-380 does not appear to modulate TNF-induced NFκB activity in this cellular model.

### Recombinant procaspase-8 phosphomimetic-Y380E mimics constitutive Src phosphorylation

**Figure 2:**
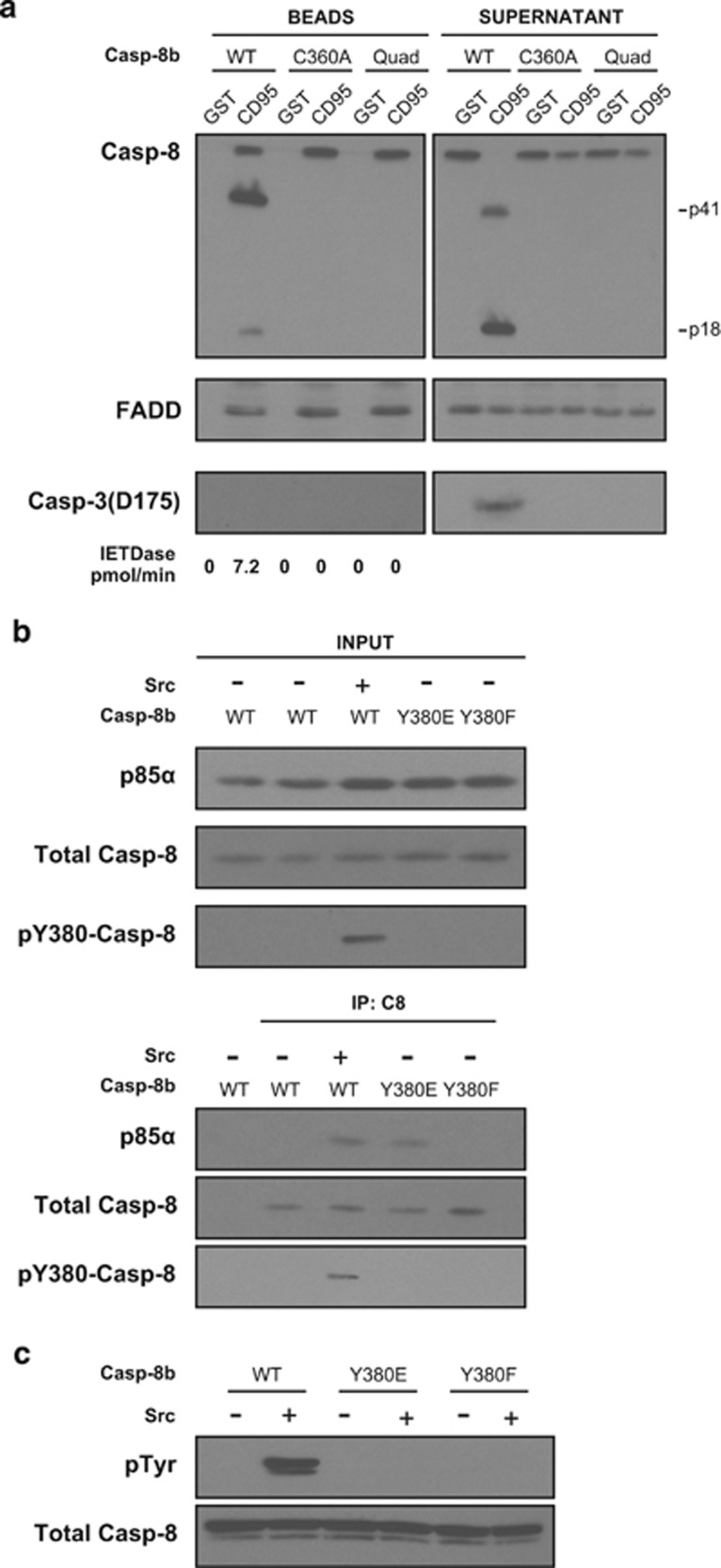
Recombinant Caspase 8 Y380E interacts with endogenous p85α. (**a**) CD95-IcD pull-downs from Jurkat caspase-8 null lysates using purified recombinant Casp-8b (WT, C360A or non-cleavable quadruple mutant (Quad)). GST beads alone were used as a control to rule out non-specific binding. Beads and supernatant were immunoblotted for FADD, caspase-8 and active caspase-3 subunits (Asp175). Note that caspase-3 was not detected in the Bead fraction post-incubation. (**b**) Lysates from Jurkat caspase-8 null cells reconstituted with purified recombinant caspase-8 with Tyr (WT), Glu or Phe at amino acid 380 were immunoprecipitated with anti-caspase-8 antibody and protein A/G beads. Inputs and Immunoprecipitated samples were analysed by SDS–PAGE and immunoblotted for p85α, total caspase-8 or phospho-Tyr380-casp-8. (**c**) Recombinant caspase-8 WT, Y380E or Y380F was incubated in the presence or absence of Src and zVAD-FMK (100 μm) for 3 h at 37 °C and subsequently subjected to SDS–PAGE and immunoblotted for total caspase-8 or phosphorylated tyrosine residues.

In addition to its non-cell-death roles, Y380 phosphorylation has also been implicated in inhibiting CD95-induced apoptosis.^13, 14, 17^ To investigate the mechanisms underlying this inhibition, we generated bacterially expressed full-length untagged wild-type caspase-8b (Casp-8b). Importantly, the resulting purified recombinant protein possessed the N-terminal DEDs so as not to preclude any contributory role that these domains may have in modulating caspase-8 function and/or activity. To confirm the activity of the purified full-length protein, we reconstituted a functional *in vitro* DISC in cell lysates using glutathione beads conjugated to the intracellular domain (IcD) of CD95 fused to glutathione S-transferase (GST).^3^ When added to caspase-8 null Jurkat cell lysates with recombinant wild-type Caspase-8b (Casp-8b-WT), the GST-CD95-IcD selectively recruited endogenous FADD and subsequently recombinant Caspase-8b (Figure 2a). This resulted in full activation of the caspase zymogen and generation of the characteristic p41 cleavage fragment (caspase-8b prodomain and large catalytic subunit) and p18 (large catalytic subunit alone), which was subsequently released into the supernatant (Figure 2a). In addition, mutation of the active site cysteine (Casp-8b C360A) or mutation of the four autoproteolytic cleavage sites (Casp-8b Quad) prevented processing of recombinant caspase-8b at the reconstituted DISC. Next, caspase-8 null Jurkat cell lysates were reconstituted with recombinant caspase-8b containing either the Y380E or Y380F mutation or wild-type caspase-8b, which had been *in vitro* phosphorylated with Src tyrosine kinase and immunoprecipitated with caspase-8 antibody. The complexes were then analysed by SDS–PAGE and western blotting, which showed that p85α exclusively co-immunoprecipitated only when recombinant caspase-8b was *in vitro* phosphorylated at tyrosine-380 (Figure 2b, lane 3) or when mutated to glutamic acid (Figure 2b, lane 4), indicating that this mutation mimicked constitutive phosphorylation, as shown previously in the overexpression experiment (Figure 1). Crucially, neither non-phosphorylated wild-type nor the unphosphorylatable Casp-8b-Y380F variant formed a complex with p85α, indicating that this requires specific interaction with charged residues at Y380. To rule out the possibility that other tyrosine residues were phosphorylated by Src, immunoblots were also performed on Casp-8b-WT, -Y380E and -Y380F variants incubated with Src and subsequently probed with a pan-phospho-tyrosine antibody (Figure 2c). This showed that only wild-type procaspase-8 was phosphorylated by Src, as neither the Y380E nor Y380F mutants exhibited a reaction with the phospho-tyrosine antibody.

### Caspase-8 phosphorylation inhibits DISC-mediated caspase-8 cleavage

**Figure 3:**
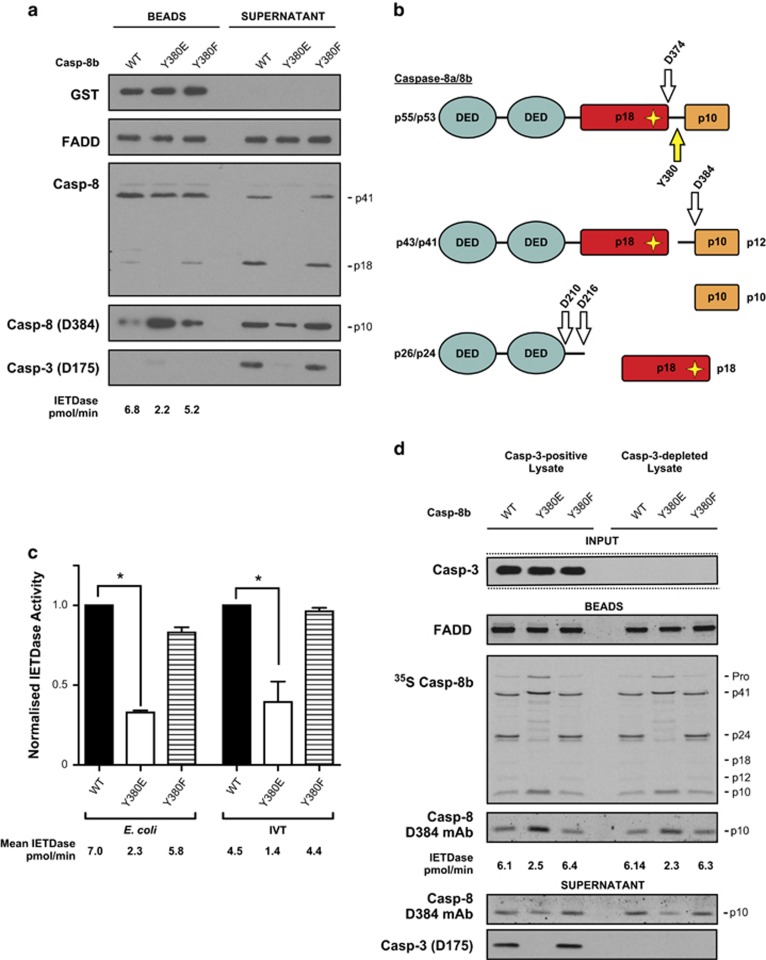
Caspase-8 phosphorylation impedes DISC-mediated maturation of procaspase-8. (**a**) CD95-IcD pull-downs from Jurkat caspase-8 null lysates using phosphorylation site WT, Y380E or Y380F mutants of purified recombinant Casp-8b derived from *E.* c*oli*; Beads and supernatant were immunoblotted for GST, FADD, caspase-8 cleavage fragments and active caspase-3 (Asp175). Note that caspase-3 was not detected in the Bead fraction post-incubation. (**b**) Putative cleavage fragments derived from procaspase-8 are shown along with the DED and starred active site Cysteine (C360). (**c**) Bead-associated IETDase activity was measured for each caspase-8 variant derived from *E.* c*oli* or IVT and normalized against the wild-type value (nominally given the value of 1). Data are mean+s.e.m. of *n*=3, *P<0.05. (**d**) CD95-IcD pull-downs from Jurkat caspase-8 null lysates and Jurkat caspase-8 null lysates immuno-depleted for caspase-3 were reconstituted with IVT-derived ^35^S-labelled recombinant procaspase-8b variants (50 μl) and analysed by SDS–PAGE and autoradiography. Beads were analysed for FADD and caspase-8 alongside IETDase activity and supernatants for active caspase-8 and caspase-3.

We next evaluated the role of caspase-8 Y380 phosphorylation in modulating DISC activation and enzymatic activity by introducing the Casp-8b-Y380E and -Y380F mutants into the *in vitro* reconstituted CD95 DISC model. In caspase-8 null Jurkat cell lysates, the Casp-8b-Y380F mutant behaved similarly to the wild type and was processed to the canonical p18 and p10 fragments (Figure 3a, lane 3) and thus subsequently activated the downstream effector caspase, caspase-3, in the corresponding supernatant fraction. However, the Casp-8b-Y380E mutant was only processed to the p41 fragment with no detectable p18 present at either the DISC or in the supernatant (Figure 3a, compare lanes 2 and 1). Interestingly, cleavage at D384 was not blocked but the majority of the resultant p10 subunit was retained within the DISC with only small amounts released into the supernatant (Figures 3a and b).

To confirm that the result obtained was not restricted to bacterially expressed recombinant procaspase-8, we repeated the experiments in caspase-8 null Jurkat cell lysates using procaspase-8 generated *via* an eukaryotic *in vitro* transcription/translation (IVT) system as described previously.^3^ Additionally, this system also permits the incorporation of ^35^S-labelled methionine into the protein and thus allows for subsequent tracking of all the cleavage fragments by autoradiography. The results obtained with this technique were in agreement with those obtained with bacterially produced procaspase-8 and further show a reduction in both p12 and p24 fragments with the Y380E mutant (Supplementary Figure S1 and Figure 3c). Furthermore, this finding was recapitulated in caspase-8 null lysates that had been additionally immuno-depleted of caspase-3, thus ruling out the possibility that feedback by this effector caspase is responsible for the reduction in IETDase activity and caspase-8 cleavage pattern observed (Figure 3d). Together, this suggests that caspase-8-Y380 phosphorylation prevents cleavage at D210, D216 and D374 as well as markedly reducing DISC IETDase activity by ~70% compared to that obtained with wild-type caspase-8 (Figure 3c).

### Y380 phosphorylation inhibits procaspase-8 inter-dimer processing at the CD95 DISC

**Figure 4:**
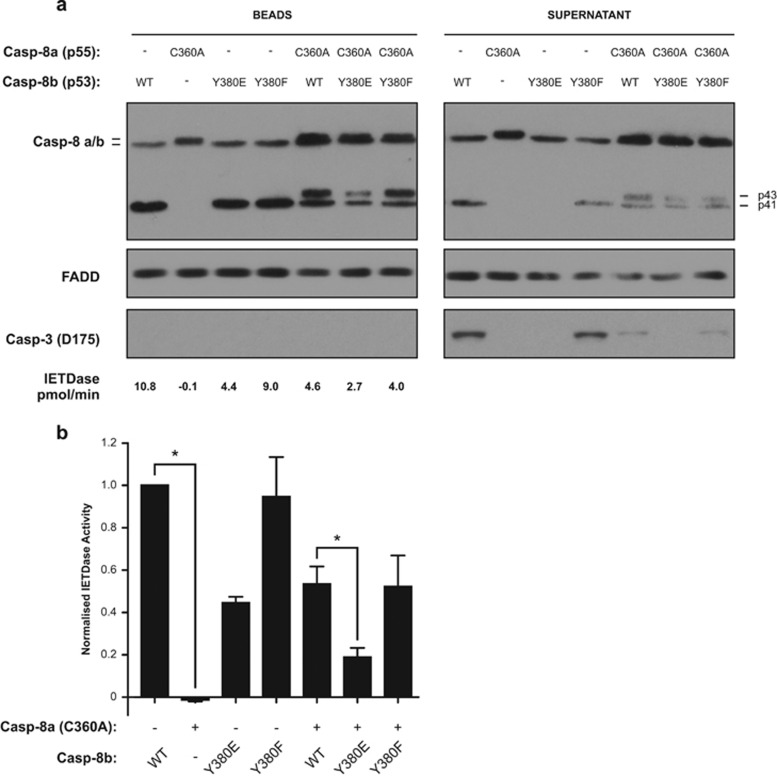
Y380 phosphorylation inhibits procaspase-8 inter-dimer processing at the CD95 DISC. (**a**) CD95-IcD pull-downs from Jurkat caspase-8 null lysates using active site WT Casp-8b (WT, Y380E or Y380F) or C360A mutants of recombinant Casp-8a, either singly or in a 1:1 ratio. Beads and supernatant were analysed for recombinant caspase-8, FADD and lysates for active caspase-3 (Asp175) and IETDase bead activity measured. (**b**) Graphical representation of IETDase values from (**a**). Normalized against wild-type caspase-8b alone. Data are mean±s.e.m., *n*=3, *P<0.05.

As the Y380E mutation abrogated DISC IETDase activity (Figure 3), we next investigated whether this mutation also affected the ability of caspase-8 to cleave its dimer partner within the DISC. To study this, an active site mutant of procaspase-8a (Casp-8a-C360A) was added to caspase-8 null Jurkat cell lysates in combination with wild-type procaspase-8b or the Casp-8b-Y380 mutants. As predicted, when introduced alone the Casp-8a-C360A mutant was recruited to the CD95 DISC, but was not cleaved, and did not exhibit any IETDase activity (Figures 4a and b). However, when the Casp-8a-C360A mutant was reconstituted with either the Casp-8b-WT or the -Y380F mutant, both procaspase-8b and the active site mutant procaspase-8a were processed to p41/p43 fragments respectively, indicating that both the WT and Y380F variants can cleave their dimer partner at the DISC. However, when the Casp-8b-Y380E mutant was supplemented in combination with the catalytically inactive procaspase-8a variant, reduced processing of both procaspase-8a and -b to their p43/p41 forms respectively was observed in addition to reduced downstream cleavage of procaspase-3 (Figure 4a). This observation suggests that, while phosphorylation of the Y380 position does not completely inhibit the cleavage of a partner caspase-8 molecule at the CD95 DISC, it does however significantly inhibit this critical step.

### Caspase-8 phosphorylation attenuates CD95-mediated apoptosis in SH-SY5Y cells

**Figure 5:**
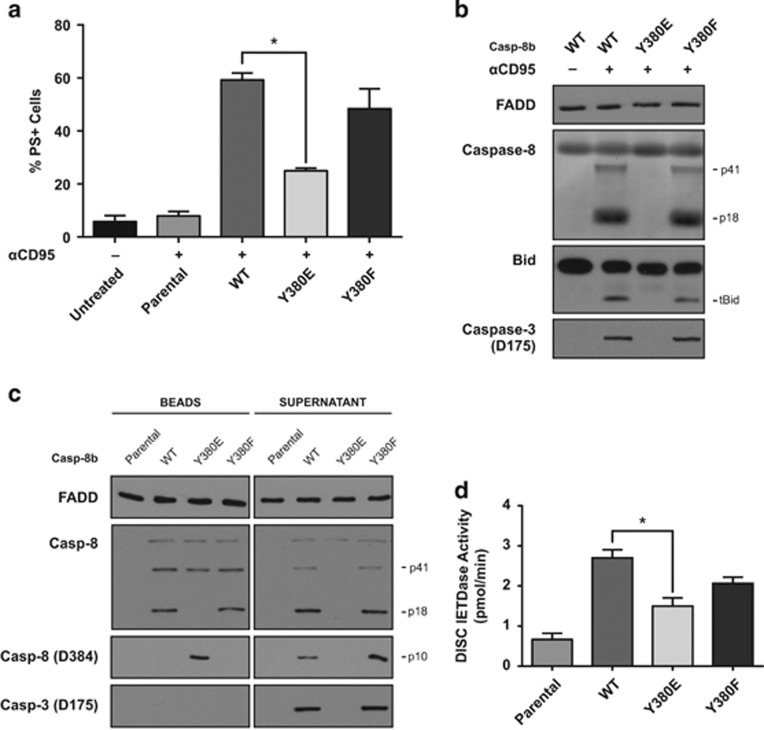
Caspase-8 phosphorylation inhibits apoptosis by impeding native CD95 DISC function. (**a**) Apoptosis in SH-SY5Y cell clones stably expressing WT or mutant procaspase-8b after 4 h exposure to anti-CD95 (αCD95; 400 ng/ml) (mean+s.e.m., *n*=3). (**b**) Immunoblots of whole-cell lysates following anti α-CD95 treatment using antibodies raised against caspase-8, FADD, BID and caspase-3. (**c**) CD95 DISC Pulls from SH-SY5Y cells expressing phosphorylation site Casp-8b WT, Y380E or Y380F mutants. Beads and supernatant were immunoblotted for FADD, caspase-8 cleavage fragments and active caspase-3. (**d**) IETDase activity of CD95 DISC Pulls from SH-SY5Y cells expressing phosphorylation site Casp-8b WT, Y380E or Y380F mutants. Values are mean+s.e.m., *n*=3, *P<0.05.

To further investigate whether caspase-8 phosphorylation has a role in mediating cellular life/death decisions, the neuroblastoma re-expression model described in Figure 1 was again utilized to explore the influence of caspase-8 phosphorylation on CD95-mediated cell death. Thus, in the absence of endogenous caspase-8, SH-SY5Y cells are resistant to CD95-induced apoptosis, as evidenced by the lack of Annexin-V binding in the parental cell line following anti-CD95 treatment (Figure 5a). However, when wild-type procaspase-8b is re-expressed, the cells become highly sensitive to anti-CD95, with approximately 60% of cells displaying an apoptotic phenotype after 4 h of treatment. Conversely, in cells expressing equivalent levels of the phosphomimetic Casp-8b-Y380E, apoptotic cell death is greatly reduced with only ~23% of cells undergoing apoptosis (Figures 1b and 5a).

Analysis of immunoblots of anti-CD95-treated cells revealed that caspase-8 cleavage was not present in the phosphomimetic expressing lines (Figure 5b), which correlated with complete abrogation of downstream caspase activation, as evidenced by the absence of caspase-3 processing or cleavage of Bid to tBid. The lack of Bid cleavage would further inhibit apoptotic signalling as it would prevent engagement of the intrinsic mitochondrial apoptotic pathway. These data clearly indicate that caspase-8 tyrosine-380 phosphorylation can have a critical role in regulating life/death decisions within the cell, particularly in type II cells where the mitochondrial amplification loop is required for CD95-induced apoptosis.

To investigate whether this reduced rate of apoptosis was a consequence of reduced DISC formation and/or activity, native CD95 DISC pull-downs were performed. In agreement with the recombinant caspase-8 data *in vitro* (Figures 2 and 3), the Casp-8b-Y380E expressing cells did not generate the catalytic p18 fragment and had reduced cycling of the p41 fragment as well as retention of the p10 subunit within the DISC (Figure 5c). Consequently, downstream processing of caspase-3 was not evident, thus explaining the reduction in apoptotic cell death. Furthermore, there was a significant reduction in CD95 DISC-associated IETDase activity in the caspase-8 Y380E expressing cells when compared with the wild-type expressing cells (Figure 5d). The apparent contradictory finding that there is measureable IETDase activity, but no observable cleavage of procaspase-3, may be due to the fact that phosphorylation of caspase-8 potentially causes the active site to be sterically blocked to large protein substrates such as caspase-3, yet still allow access to small moieties such as Ac.IETD-AFC.

### Caspase-8 Y380 phosphorylation attenuates CD95 DISC activity in cell lysates but not in a lysate-free DISC reconstitution system

**Figure 6:**
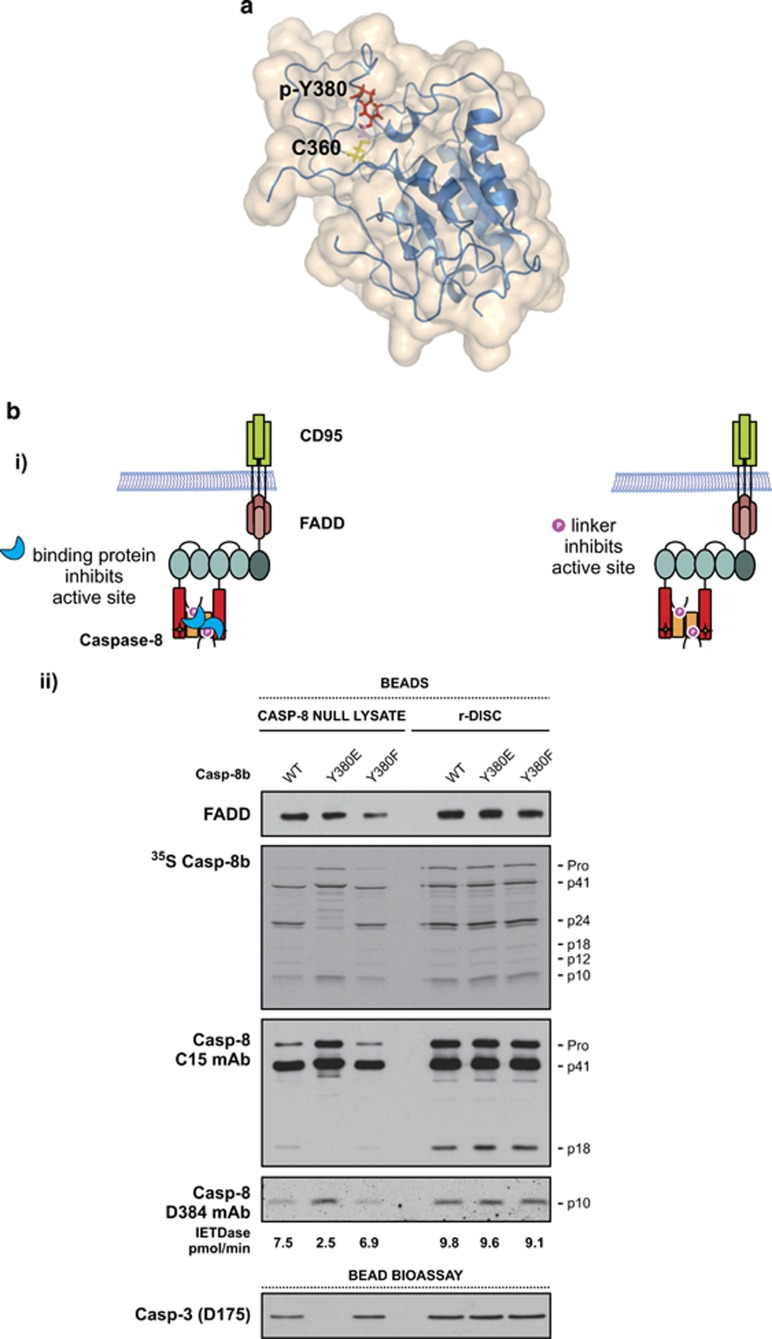
Caspase-8-Y380 phosphorylation attenuates CD95 DISC activity in cell lysates but not in a lysate-free system. (**a**) Model of caspase-8 derived from a solution structure of the monomeric unprocessed catalytic domain of the caspase-8 zymogen (PDB: 2K7Z). The active site cysteine 360 has been modelled to replace the original structure's alanine residue, highlighted in yellow; tyrosine-380 and modelled phosphate are highlighted in red and pink, respectively. (**b**) (i) Schematics depicting two possible inhibition mechanisms, either an unknown binding partner interacts with the phosphorylated tyrosine, thereby sterically hindering the active site or causing a conformational change, and/or the phosphorylated linker directly interacts with and inhibits the caspase-8 active site. (ii) CD95-IcD pull-downs from Jurkat caspase-8 null cell lysates or the lysate-free fully reconstituted DISC (r-DISC) with CD95-IcD, r-FADD (5 μg), both supplemented with ^35^S-labelled recombinant procaspase-8b. Beads were analysed for FADD, caspase-8 and active caspase-8 (D384). DISC activity was further bioassayed for r-procaspase-3 (C163A) cleavage.

We hypothesize that caspase-8 activity could be inhibited *via* one or more mechanisms following Y380 phosphorylation. First, the phosphorylated linker may directly interact with, and inhibit the active site of the enzyme. As yet, no structural data are available for full-length procaspase-8; as a result, it is not currently known whether the linker region structurally influences the prodomain of the protein or *vice-versa*. However, one set of solution structures is available for the catalytic domains.^17^ By molecular modelling of phospho-Y380 onto one of these structures we found a potential close interaction between the putative phosphate moiety and the catalytic triad (Figure 6a). This interaction could disrupt or partially block the active site and hence impair caspase-8 catalytic activity (Figure 6b i). Alternatively, the phosphate moiety could recruit other proteins that may sterically hinder the active site and/or induce a conformational change in caspase-8, thus preventing both its complete autoactivation and cleavage of secondary substrates such as caspase-3 (Figure 6b i). To distinguish between these two possibilities, the fully reconstituted CD95 DISC (r-DISC), comprising only recombinant CD95-IcD, recombinant FADD and recombinant procaspase-8, was assembled *in vitro*.^3^ We reasoned that if the phosphate moiety alone was causing the inhibition then the same characteristic cleavage pattern would be observed in both cell lysates and the lysate-free DISC reconstitution system. However, in the lysate-free system, and thus in the absence of potential binding partners, the Y380E mutant behaved similarly to the wild-type variant and permitted cleavage of the effector procaspase-3 in an *in vitro* bioassay (Figure 6b ii, compare lanes 4 and 5). Together, these findings suggest that an additional factor present in the cell lysate is required for Y380 phosphorylation-mediated inhibition of procaspase-8 complete maturation and activation at the DISC.

## Discussion

Recent studies have shown that outside of its canonical role in cell death, caspase-8 has additional functions in migration, adhesion^12^ and endosome maturation.^13^ In these scenarios, Src-mediated phosphorylation of caspase-8 has been identified as a potential key switch in determining whether it functions in an apoptotic or non-apoptotic role.

Previous studies indicated that Src phosphorylates Tyrosine-380, a residue found in the linker region between the two catalytic subunits of caspase-8 (Figure 1a) and this phosphorylation modulates its function.^14^ Phosphorylation at this site results in increased resistance to CD95-mediated apoptosis, reportedly due to the reduced cleavage of procaspase-8.^14^ However, the mechanism underlying this increased resistance has not been characterized in the context of procaspase-8 activation within the DISC. In the current study, we have investigated the underlying mechanism using: (1) an *in vitro* CD95 DISC model consisting of full-length recombinant procaspase-8 and its mutants to mimic constitutive phosphorylation; (2) native DISCs isolated from anti-CD95-activated cells. Our findings reveal that phosphorylation of Y380 results in the inhibition of caspase-8 function by blocking the complete maturation and activation of this key molecule within the DISC, but only in the presence of additional cell lysate components.

**Figure 7:**
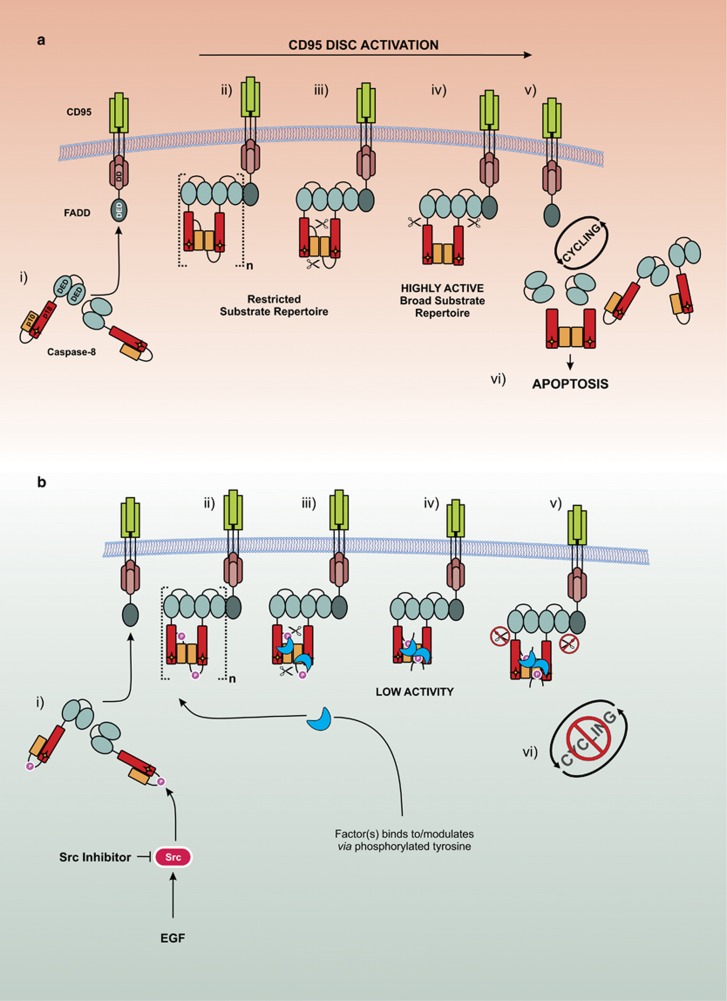
Model depicting proposed mechanism of caspase-8 inhibition by Tyr380 phosphorylation. (**a**) Canonical procaspase-8 activation. (i) cytoplasmic procaspase-8 is recruited to the CD95/FADD complex at the cell-surface membrane leading to (ii) dimerization and DED-mediated caspase-8 chain assembly. This is followed by (iii) autoproteolytic cleavage at D384 to form a (iv) highly active DISC, subsequently the active catalytic subunits are then (v) autoproteolytically cleaved at D210/216 and released from the DISC to allow for (vi) further cycling and ultimately cell death. (**b**) Y380 phosphorylation-mediated inhibition of procaspase-8. (i) cytoplasmic procaspase-8 is phosphorylated by Src and later recruited to the CD95/FADD complex leading to (ii) dimerization, procaspase-8 DED chain assembly and binding of an unknown partner to the phosphorylated tyrosine residue leading to steric hindrance and/or a conformational change of the catalytic site. This is followed by (iii) autoproteolytic cleavage at D384 to form a (iv) complex with low enzymatic activity and (v) prevent further autoproteolytic cleavage and release of the p18 subunit therefore blocking (vi) cycling of procaspase-8 at the DISC.

Our data lead us to propose a model (Figure 7) in which caspase-8 is phosphorylated at Y380 by Src in response to external cues such as EGF stimulation or an increased requirement for cell migration (Figure 7b i). This phosphorylation event does not prevent recruitment of procaspase-8 to the CD95 DISC, as evidenced by the ability of the phosphomimetic (Y380E) to bind to FADD and form a DISC (Figure 3a); it is therefore also unlikely to affect DED-mediated procaspase-8 chain assembly.^16, 18^ Nor does it prevent homodimerization, as procaspase-8 Y380E is still able to cleave its dimer partner at the complex (Figure 4). However, in the presence of cell lysate, but not in the lysate-free fully reconstituted r-DISC, subsequent steps are guided by the presence of the phosphorylated residue. First, in both phosphorylated and non-phosphorylated caspase-8 containing complexes, caspase-8 is auto-processed at D384 to separate the p10 catalytic subunit (Figures 7a/b ii and 3a). At this stage, the conventional, non-phosphorylated, DISC is highly active and displays a broad substrate repertoire as demonstrated by cleavage of two of its key substrates, Bid and procaspase-3 (Figures 7a iv and 5). By comparison, the phosphorylated DISC displays greatly reduced IETDase activity, down to approximately one-third of wild type (Figure 3), and a limited substrate repertoire and is thus unable to process procaspase-3 (Figures 7b iv, 3 and 5).

In the canonical DISC, where caspase-8 remains unphosphorylated, this is followed by the release of the p18 subunit from the DISC (Figure 7a v), due to further cleavage at D210 and D216. However, this step is completely blocked by Y380 phosphorylation (Figure 7b v) as shown by the lack of the p18 species in the supernatant fraction (Figures 3, 4, 5), therefore preventing full activation of caspase-8; an event that occurs independently of any caspase-3 feedback (Figure 3d). Moreover, the phosphorylation event precludes further cycling of caspase-8 at the DISC (Figure 7b vi), thus preventing further procaspase-8 molecules from being recruited to the complex and limiting the extent of DISC activity by also reducing the cytosolic levels of mature caspase-8 catalytic subunits, that is, p10 and p18, available for downstream effector caspase activation (Figure 3).

In our model system, we did not observe any effect of caspase-8 Y380 phosphorylation on NFκB signalling upon TNFα stimulation. However, other death-inducing ligands such as TRAIL are also capable of stimulating this pathway; it would therefore be interesting to investigate whether the signalling outcomes of these other death ligands are influenced by caspase-8 phosphorylation. Furthermore, while we have only investigated Y380 phosphorylation, there remain other caspase-8 phosphorylation sites such as Tyrosine-293/448 and Serine-287/305/347,^8, 9, 10, 11^ which could further influence cell fate by modulating life/death decisions.

Multiple cancers display genetic alterations that lead to impaired caspase-8 function and/or upregulation of Src family kinases^19, 20, 21^ thereby increasing resistance to death receptor-induced apoptosis. Indeed, a concomitant increase in both Src and caspase-8 phosphorylation and associated resistance to Fas-induced apoptosis is observed in colon cancer.^14^ Furthermore, Src inhibition can sensitize colon cancer cells to apoptosis^22, 23^ and since we observed that Y380 phosphorylation also has a role in cell migration, Src inhibition may further help prevent tumour metastasis. More recently, some breast cancer cell lines have been shown to activate Src in response to TRAIL treatment^24^ and it is thought this may be a mechanism of acquired TRAIL resistance, as a concomitant increase in caspase-8 phosphorylation was observed. Moreover, Lyn-mediated phosphorylation of Y380 was found to promote resistance to apoptosis in chronic lymphocytic leukaemia due to the formation of an inactive procaspase-8 homodimer.^25^ In addition, tumours displaying intrinsic Y380 phosphorylation such as chronic lymphocytic leukaemia and colon cancer could potentially be treated by inhibiting Src activity *via* medicinal intervention, thus removing the caspase-8 Y380-mediated block on cell death and potentially sensitizing them to apoptosis-inducing drugs. The finding that other, as yet unidentified, factors are required for Y380 phosphorylation-mediated inhibition of apoptosis provides additional potential therapeutic targets; identification of these factors is therefore an interesting avenue for future investigation.

Although Y380 is found in the putative linker region in other primates, *Xenopus* and zebrafish, it is absent in rodents, suggesting that in these species this regulatory mechanism is absent. Therefore, if modulation of caspase-8 function *via* Src inhibition is to be exploited in the treatment of human carcinomas, care must be taken when choosing appropriate pre-clinical model systems to explore new therapeutic agents.

## Materials and methods

### Materials

Media and serum were purchased from Invitrogen (Paisley, UK). Antibodies were sourced as follows: FADD mouse mAb (610400) was from BD Transduction Laboratories (San Jose, CA, USA); GST tag mouse mAb (71097) was from Novagen (Watford, UK); cleaved caspase-3 (Asp175) (9661) rabbit polyclonal antibody, cleaved caspase-8 (Asp384) (11G10) mouse mAb, Bid (2002) rabbit polyclonal antibody, Phospho-IκBα (Ser32) (14D4) rabbit mAb and total IκBα (44D4) rabbit mAb were sourced from Cell Signaling Technology Inc. (Hitchin, UK); caspase-8 rabbit polyclonal antibody was generated in our laboratory; caspase-8 mouse mAbs (C15 and N2) were kind gifts from Prof. PH Krammer (German Cancer Research Center, Heidelberg, Germany); the p85α and pan-Phospho-tyrosine (2C8) mouse mAbs were from Santa Cruz Biotechnology, Inc. (Middlesex, UK); Phospho (Tyr380) caspase-8 was a kind gift from Prof. D Barila (University of Rome Tor Vergata, Rome, Italy). Horseradish peroxidase-conjugated secondary antibodies were obtained from Sigma-Aldrich (goat anti-mouse; Dorset, UK) and DAKO (goat anti-rabbit; Cambridge, UK). The caspase-8 substrate, Ac-Ile-Glu-Thr-Asp-amino-4-trifluoromethyl coumarin (Ac-IETD.AFC) was purchased from MP Biomedicals (Cambridge, UK). All other chemicals were of analytical grade and obtained from Sigma-Aldrich or Fisher (Loughborough, UK).

### Vectors and constructs

Full-length caspase-8 was cloned into the *Sap*I and *Nde*I sites of pTYB1 (New England BioLabs Inc., Hitchin, UK). pCDNA3.1 caspase-8 constructs have been previously reported.^3^ Full-length caspase-8 constructs were cloned into pIRES Puro (Clontech Laboratories, Mountain View, CA, USA). Site-directed mutagenesis was performed using the QuikChange Mutagenesis kit (Stratagene, Santa Clara, CA, USA) with appropriate mutagenesis primers.

### Cell culture and transfections

Human neuroblastoma cells (SH-SY5Y, from ATCC) deficient in caspase-8 were cultured in 1:1 DMEM:Nutrient F-12 Ham supplemented with 10% fetal bovine serum and glutamine (2 mm). Human T-lymphocyte cells (Jurkat, clone A3 and caspase-8-deficient, clone I9.2; kind gifts from Prof. J Blenis, Weill Cornell Medical College, New York, USA) were cultured in RPMI and supplemented as previously described.^3^ SH-SY5Y cells were transfected using the Lipofectamine-2000 reagent (Invitrogen) following the manufacturer's instructions. Cell lines expressing caspase-8 variants were selected by flow cytometry for GFP-positive cells.

### Quantitative RT–PCR

Total RNA was extracted from SH-SY5Y cells using TRIzol reagent (Thermo Fisher Scientific, Waltham, MA, USA) according to the manufacturer's instructions. cDNA was synthesized using SuperScript III (Thermo Fisher Scientific) from total RNA. Quantitative real-time PCR was performed to quantify the expression of IL-6 using Fast SYBR Green Master Mix (Thermo Fisher Scientific) according to the manufacturer's protocol on an Applied Biosystems QuantStudio 6 Flex System (Waltham, MA, USA). As an internal control for the normalization of these genes, ribosomal protein L18 (RPL18) was used. The primer sequences and specifications are summarized below. The fold-change in TNF-treated cells relative to untreated matched controls was calculated using the ΔΔCt method. Each assay was performed in triplicate.

IL-6

Forward: 5′-CCAGGAGCCCAGCTATGAAC-3′

Reverse: 5′-CCCAGGGAGAAGGCAACTG-3′

RPL18

Forward: 5′-CCCAAGAGCCAGGATATCTACCT-3′

Reverse: 5′-TTGAATGTGGAGTTGGTTCTTCTG-3′

### *In vitro* DISC model: assembly and analysis

Caspase-8 null Jurkat cell lysates were made as described previously.^3^ For receptor pull-downs, 10 mg of cell lysate (~20 mg/ml) was incubated with 10 μg of purified GST-CD95-IcD fusions bound to glutathione Sepharose beads for 16 h at 23 °C.^26, 27^ Where indicated, caspase-8-deficient lysates were supplemented with IVT-generated r-procaspase-8 or Intein purified r-procaspase-8. Control assays were carried out using beads coated with GST alone. Bead-associated complexes were washed in PBS containing complete protease inhibitors (Roche, West Sussex, UK), released from beads by boiling in SDS sample buffer, and analysed by SDS–PAGE/western blotting. To assess caspase-8 activity, beads were resuspended in caspase assay buffer (100 mm HEPES, 10% sucrose, 0.1% CHAPS and 10 mm dithiothreitol (pH 7.0)), and cleavage of the fluorogenic substrate Ac-IETD.AFC (40 μm) was measured at 37 °C on a Wallac Victor2 fluorometer (Perkin Elmer, Waltham, MA, USA). Lysate-free, CD95 DISC reconstitutions (r-DISC) were carried out as previously described.^3^ A bioassay of DISC-associated caspase-8 activity was performed using a recombinant active site mutant of procaspase-3 (C163A) as previously described.^3^

### Generation of caspase-3 immuno-depleted lysates

Dynabeads Protein A (Invitrogen Dynal, Oslo, Norway) was washed with PBS-Tween (0.05%) and incubated with polyclonal rabbit anti-Caspase-3 antibody (MRC Toxicology Unit, UK) for 1 h at 4 °C. Beads were washed with 0.2 m Triethanolamine (TEA, pH 8.2) and resuspended in 20 mm Dimethyl pimelimidate dihydrochloride (DMP) dissolved in 0.2 m TEA and incubated for 30 min at room temperature and then 15 min with 50 mm Tris (pH 7.5). Crosslinked beads were washed with PBS-Tween before being incubated with Caspase-8 null Jurkat cell lysates for 16 h at 4 °C. The subsequent supernatant was retained and used as caspase-3-depleted caspase-8 null lysate.

### Precipitation of the CD95 DISC from SH-SY5Y cells

SH-SY5Y cells were incubated with pre-chilled media containing 400 ng/ml Apo1-3BT with 0.05 μl/ml protein A and incubated on ice for 45 min to preassemble the DISC with subsequent incubation at 37 °C for 20 min or 4 h for apoptosis measurements by Annexin-V/PI staining.^3, 28, 29^ Cells were washed twice with chilled PBS and cells lysed in DISC lysis buffer (30 mm Tris pH 7.5, 150 mm NaCl, 10% (v/v) glycerol, 1% (v/v) Triton X-100 containing complete protease inhibitor cocktail (Roche)) for 30 min on ice. Lysates were cleared by centrifugation at 15 000 *g* for 30 min and then incubated overnight with streptavidin magnetic beads at 4 °C on a daisy wheel. Beads were subsequently washed and analysed by western blotting or IETDase activity measured.

### Generation of recombinant procaspase-8 protein

Full-length recombinant procaspase-8 was produced and purified from *E. coli* ER2566 cells using the Impact Intein purification system (New England BioLabs Inc.) according to the manufacturer's instructions. IVT-generated recombinant procaspase-8 was generated as previously described.^3^

### Western blotting

SDS–PAGE and western immunoblotting were carried out as described previously.^30^

### Co-immunoprecipitaion

Cells were lysed in IP buffer (20 mm Tris pH 7.4, 150 mm NaCl, 1% NP-40) supplemented with complete protease inhibitor mixture (Roche) and centrifuged at 13 000 *g* for 10 min at 4 °C. Protein concentration was determined by Bradford assay. For immunoprecipitation, 500 μg of protein was incubated with 2 μg of mouse anti-caspase-8 antibody (BD; 551242) overnight at 4 °C. Complexes were precipitated with 25 μl of protein A/G (Dynal). Beads were washed five times with PBS, eluted in boiling Laemmli buffer and resolved by 12% SDS–PAGE.

### *In vitro* Src phosphorylation

Phosphorylation of procaspase-8 was accomplished by incubation with Src kinase (New England BioLabs Inc.) with 25-fold excess of procaspase-8 and 5-fold excess of ATP at 37 °C for 3 h in phosphorylation buffer (50 mm Tris pH 8.0, 150 mm NaCl, 0.5 mm TCEP, 20 mm MgCl_2_, 12.5 mm MnCl_2_ and 12.5 mm β-glycerolphosphate).

### Migration assays

Six-well dishes were coated with 2 μg/ml of fibronectin overnight in PBS (pH 8.0). SH-SY5Y cells were allowed to attach for 2 h at confluent density (500 000 cells/well), and then monolayers were wounded twice with a conditioned pipette tip at 90° angles to generate a ‘cross-roads' wound. Multiple regions were measured microscopically, the wound was permitted to close for 24 h, and regions were re-measured, and the mean wound closure calculated. The cross-roads assay permitted the same regions to be relocated and measured after the incubation period.

### Statistical analysis

Unless otherwise stated, data are expressed as mean+s.e.m.; data were analysed with Prism software Version 6.0 (GraphPad, La Jolla, CA, USA) using the Mann–Whitney test. Values of *P*<0.05 were considered statistically significant.
