# Supplementary material for: Caspase-8 tyrosine-380 phosphorylation inhibits CD95 DISC function by preventing procaspase-8 maturation and cycling within the complex
Source: Oncogene. 2016 Apr 25;35(43):5629–40. doi: 10.1038/onc.2016.99 (PMC5095593; doi:10.1038/onc.2016.99)
Supplement: Supplementary Information [file onc201699x1.docx]

## Supplemental Data

##
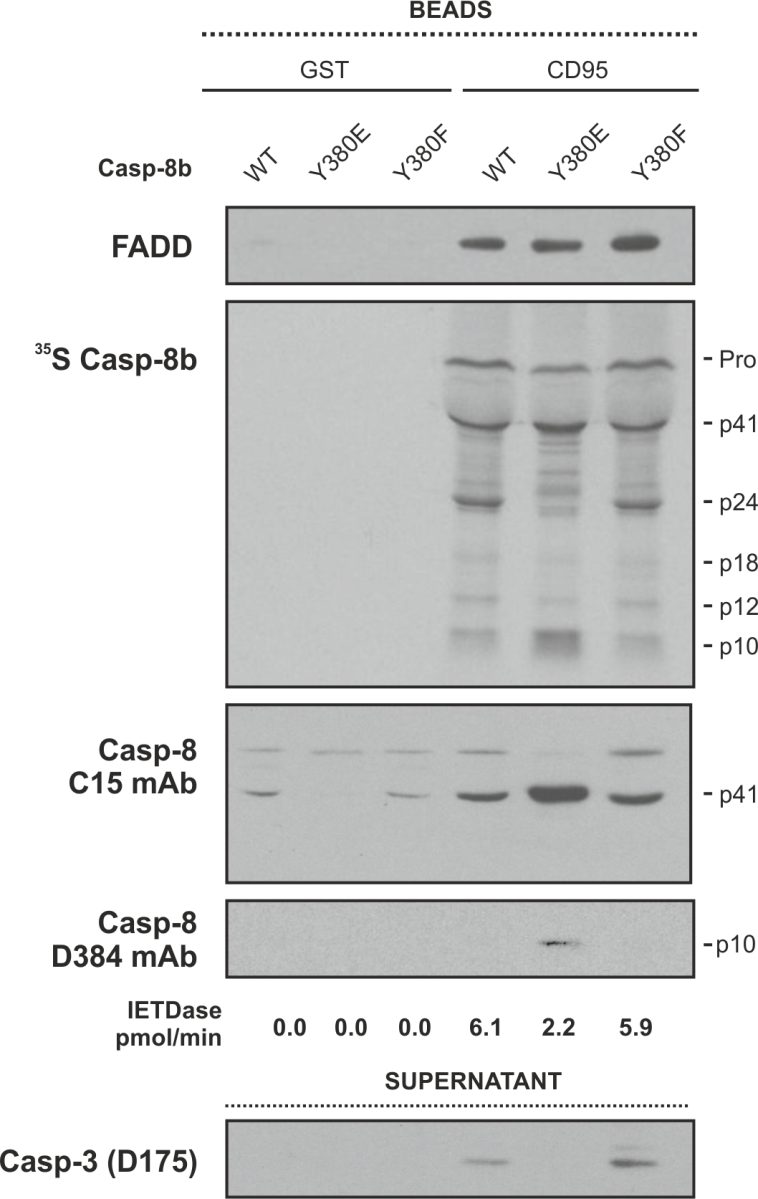


## Supplemental Figure S1

CD95-IcD pull-downs from Jurkat caspase-8 null lysates using IVT derived ^35^S-labeled recombinant procaspase-8b (50 μl) were analysed by SDS-PAGE and autoradiography alongside GST bead control.  Beads were analysed for FADD, caspase-8 and active caspase-3, alongside IETDase activity.
